# Microbial Quality and Safety of Raw Vegetables of Fiche Town, Oromia, Ethiopia

**DOI:** 10.1155/2022/2556858

**Published:** 2022-02-17

**Authors:** Birhanu Degaga, Israel Sebsibe, Tolosa Belete, Adugna Asmamaw

**Affiliations:** ^1^Salale University, Applied Microbiology, Fiche, Ethiopia; ^2^Salale University, Ecology and Systematic Zoology, Fiche, Ethiopia; ^3^Salale University, Environmental Sciences, Fiche, Ethiopia; ^4^Salale University, Botany, Fiche, Ethiopia

## Abstract

Vegetables contain vital ingredients such as minerals, phytochemicals, vitamins, and fibers, which play significant roles in human health. Consumption of fresh vegetables causes human infections and outbreaks while serving as a reservoir of several pathogens. The study evaluated the microbiological quality of raw vegetables consumed in and around Fiche town, Central Ethiopia. For the experimental study, a total of 100 samples of 5 different raw vegetables from two local markets were selected based on their commonalities for overall microbial quality in terms of aerobic mesophilic count, total coliform count, Enterobacteriaceae count, Staphylococci count, and yeast and mold levels. The highest count was aerobic mesophilic bacteria (5.7 log CFU/g) followed by Enterobacteriaceae (4.7 log CFU/g), while yeasts and molds count the least. The maximal count for aerobic mesophilic bacteria was enumerated in cabbage (6.4 log CFU/g) while the minimum was in green pepper samples (4.7 log CFU/g). Among 100 vegetable samples analyzed, 11% were contaminated by *S. aureus* which is highly prevalent in cabbage (20%), followed by lettuce (15%). In the present study, 15.0% of vegetable samples were positive for *Salmonella* and detected in all vegetable types.

## 1. Introduction

The consumption of vegetables has enormously increased because of their nutritive values in daily dietary intake. Vegetables are important sources of many minerals and vitamins too. The Australian dietary guidelines recommend that an adult human should eat at least five kinds of vegetables every day [1]. Demand for the consumption of vegetables is on the rise since the fresh production of vegetables provides biologically active compounds such as phenolics, anthocyanins, and antioxidants that aid in better health [2] and have a prominent role to play in the prevention of heart diseases, cancer, diabetes, obesity, and micronutrient deficiencies (zinc, iron, etc.) mainly in developing countries [3]. In addition, there are broad product development opportunities because vegetables are a handy snack food and are easily carried. Currently, farmers and local public markets are the chief retailers of raw vegetables [4].

But, the consumption of fresh vegetables causes human infections and outbreaks while serving as a reservoir of several pathogens [5–8] Moreover, Halablab et al. [9] indicated that there is increased consumer demand for fresh, natural, and organically cultivated products, devoid of microorganisms. This necessitates to handle the product with care before it reaches the consumers. Enteric pathogens such as *Escherichia coli* and *Salmonella* are among the greatest concerns during food-related outbreaks. Several cases of typhoid fever outbreaks have been associated with eating contaminated vegetables grown in or fertilized with contaminated soil or sewage [4]. It has been indicated that various bacterial pathogens, including *Salmonella*, *Shigella*, *Campylobacter, E. coli* O157 : H7, *Listeria monocytogenes*, and *Staphylococcus aureus*, are the contaminants for most vegetables [8, 10–13].

The presence of aerobic mesophilic bacteria found in food is one of the microbiological indicators for food quality [14]. While indicating the existence of favorable conditions for the multiplication, coliform bacteria have long been used as indicator organisms that reflect the general microbiological condition of foods and water. It is a potential public health risk [15, 16]. *Staphylococcus aureus* induces food poisoning through its enterotoxins that are frequently responsible for food-borne illness outbreaks [4].

In different parts of Ethiopia, vegetables are being widely cultivated traditionally by rural farmers for several decades. The farmers provide vegetables to the local market. But the absence of well-ventilated storage, lack of pre and postharvesting practice at marketplaces, and inappropriate transportation techniques have been major constraints of market quality [17]. In these conditions, potential physical damages and contamination of vegetables with animals and human feces become undoubtedly possible before consumption [10]. Vegetables can also be contaminated during transportation, selling, storage, and after purchase by consumers [14]. Fresh vegetables can be contaminated at any point in the production and market chain, posing a potential food safety problem because they are likely to be consumed raw [18, 19]. In addition, prevention mechanism failure may lead to a large economic loss following an outbreak [20]. Thus, it is rational to assess the safety of these vegetables in consideration to the consumers' safety.

## 2. Materials and Methods

### 2.1. Study Design, Study Population, and Data Source

Based on cross-sectional study design, 120 vegetable cultivators and sellers were selected on sites from which vegetable samples were also purchased to get reliable information. The samples were collected from Komando and Fitche sites. These different sites were selected to represent the whole vegetables sold in Fitche town. The study periods were covered from January 2020 to October 2020. Primary data were generated from the survey, and standard laboratory experiments were used to analyze vegetable samples collected from markets and shops in Fitche town.

### 2.2. Data Collection

Vegetable cultivators and sellers were surveyed using a systematic random sampling method. A survey was conducted using structured questionnaires from randomly selected vegetable cultivators, sellers, and consumers from which the vegetable samples were purchased.

### 2.3. Sample Collection

A total of 100 fresh vegetable samples were collected from two different sites of Fiche town. Taking samples from different places allowed products that are commonly available to consumers to be sampled which makes the results of this study more representative. Based on their commonalities, tomato (*Solanum copersicum)*, cabbage (*Brassica oleracea*), green pepper (*Capsicum annuum*), lettuce (*Lactuca sativa*), and carrot (*Daucus carota*) were selected to be tested. The samples comprised of 20 each of selected commodities and were collected using sterile plastic bags. The samples were sent for analysis within 6 hours of the collection.

### 2.4. Sample Preparation

Packaging bags were aseptically opened using a sterile stainless steel knife, and 25 g portions were weighted and shaken in 225 ml of sterile 0.1% (w/v) buffered peptone water for three minutes letting the samples homogenized [21]. Total coliform count, aerobic mesophilic count, Enterobacteriaceae count, Staphylococci count, yeast, and mold were enumerated from the homogenate of the samples prepared.

### 2.5. Total Aerobic Mesophilic Count

The total viable aerobic mesophilic count was determined by plate count using the standard plate count agar (PCA) medium. Serial dilutions of the samples were made in 0.1% buffered peptone water; 0.1 ml from each dilution (10^−1^ to 10^−7^) was pipetted and spread plated on a standard presolidified plate count agar medium and incubated at 32°C for 72 hours. After incubation, plates with colonies from 30 to 300 were counted [21].

### 2.6. Total Coliform Count

A 0.1 ml of homogenate from 10^−1^–10^−5^ dilution was pipetted and spread on violet red bile agar (VRBA). Total coliforms of all vegetable samples were counted on VRBA after incubating plates at 35°C for 18–24 hours. Red to pink colonies, surrounded by precipitated bile, were counted as coliforms [22].

### 2.7. Enterobacteriaceae Count

To count the members of Enterobacteriaceae, 0.1 ml of 10^−1^–10^−5^ serial dilution of the vegetable samples were spread plated on MacConkey agar and incubated at 32°C for 24 hours. All purple colonies were counted as members of Enterobacteriaceae [22].

### 2.8. Staphylococci Count

For Staphylococci, the count was determined by mannitol salt agar (MSA). MSA was surface plated with 0.1 ml of the homogenate from 10^−1^–10^−5^ and incubated at 32°C for 36 hours. Then, golden yellow color colonies were aseptically picked and purified. A coagulase test was conducted to identify *Staphylococcus aureus* [23].

### 2.9. Yeast and Mold Counts

The count of yeasts and molds was determined by direct plate count using potato dextrose agar (PDA) supplemented with 0.1 g chloramphenicol. About 0.1 ml of the homogenate from 10^−1^ dilution was spread plated on PDA that contains chloramphenicol. The plates were incubated at 25–28°C for 3–5 days. After incubation, yeasts and molds were counted separately. Smooth (nonhairy) colonies without an extension at the periphery (margin) were considered and counted as yeasts. Hairy colonies with extension at the periphery were counted as molds [22].

## 3. Results

### 3.1. Sociodemographic Information

A total of 120 cultivars, sellers, retailers, and consumers were interviewed in which majority (90%) were from urban areas. A significant number (60%) of the respondents were consumers. Regarding educational status, majority (65%) of them were beyond secondary education. Regarding religion status, 93.3%, 5.8%, and 0.8% of the respondents were Orthodox, Protestant, and Muslim, respectively (Table 1).

### 3.2. General Sanitary Conditions

Regarding diseases caused by consuming contaminated vegetables, 85% of the respondents have such information. Majority (65%) had health problems due to the consumption of contaminated vegetables. Concerning how fresh vegetables reach the market, 45%, 25%, 20%, and 10% were transported using a car, back of humans, cart, and horse/donkey, respectively. Majority (53%) of vegetables were transported to the market using sac. Regarding the arrangement of vegetables in the market, 56% were well organized without close contact. Moreover, 57.5% of vegetable sellers have used shade to protect vegetables from physical damage due to strong sunlight and others (Table 2). Regarding time, taking fresh vegetables left with sellers/retailers, on the one hand, 40% of them responded in about 3 days before totally sold to the consumer. On the other hand, 50% of them did not add food-grade chemicals such as vinegar to raw-consumed vegetables. Moreover, only 45% of the consumers used a refrigerator to store fresh vegetables (Table 2).

### 3.3. Consumption Habit

**Figure 1:**
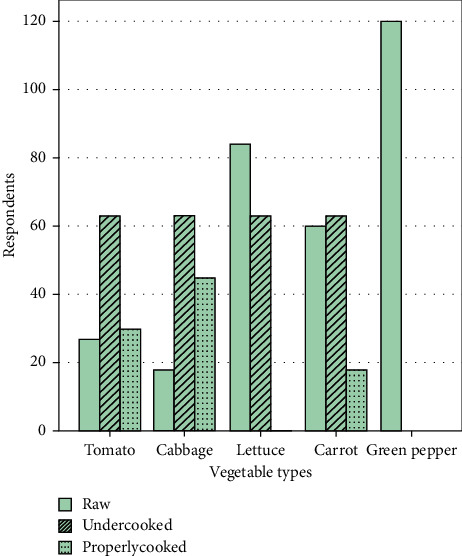
Frequently consumed vegetables in Fitche town, Central Ethiopia, 2020.

Regarding the consumption habit of consumers, majority preferred to consume vegetables as raw, specifically green pepper, carrot, and lettuce. However, tomato and cabbage were preferred to be consumed after cooking (Figure 1).

### 3.4. Microbiological Count of Raw Vegetables

Table 3 shows that the highest count was aerobic mesophilic bacteria (5.7log CFU/g) followed by Enterobacteriaceae (4.7 log CFU/g). But, the least was yeasts and mold count (2.3 log CFU/g and 2.0 log CFU/g), respectively. The maximal count for aerobic mesophilic bacteria was enumerated in cabbage (6.4 log CFU/g) while the minimum was in green pepper samples (4.7 log CFU/g). Similarly, the maximum Enterobacteriaceae and Staphylococci count were recorded in cabbage (5.7 log CFU/g and log 5.3 CFU/g) while the minimum was in carrot samples (4.2 log CFU/g and 3.3 log CFU/g), respectively (Table 3). There was a statistically significant difference (*P* < 0.05) among the mean counts of all the microbial groups in the vegetable samples.

**Figure 2:**
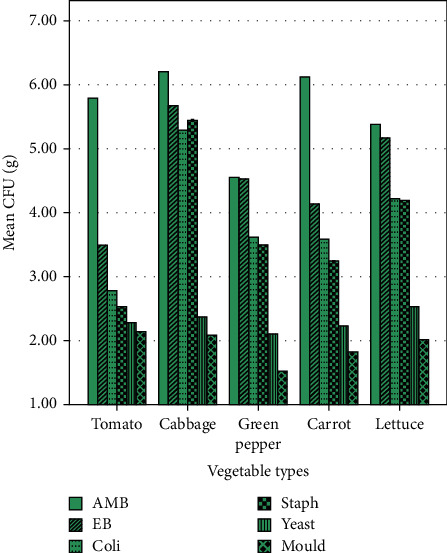
Microbiological count of raw vegetables collected from Komando site, Fitche town, Central Ethiopia, 2020.

**Figure 3:**
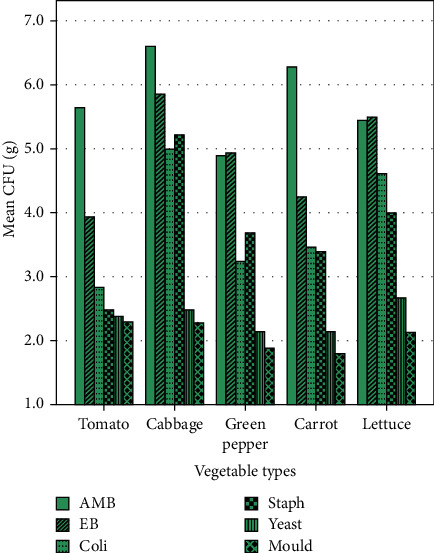
Microbiological count of raw vegetables collected from Fitche site, Fitche town, Central Ethiopia, 2020.

The aerobic mesophilic count of cabbage and carrot in both Komando and Fitche sites was higher (>6 log CFU/g). Similarly, the Enterobacteriaceae count of cabbage and lettuce is higher in both sites (>5 log CFU/g). The Staphylococci count was the lowest in tomato (<3 log CFU/g) in both sites (Figures 2 and 3).

Accordingly, the aerobic mesophilic count of tomato samples was between 5 and 6.9 log CFU/g. Similarly, all (100%) of cabbage samples had Enterobacteriaceae counts between 5 and 6.9 log CFU/g. Over 40% of vegetable samples had coliforms ≥ 4 log CFU/g. However, in all the vegetable samples, Staphylococci count was between 2 and 2.9 log CFU/g. The yeasts and molds were mostly between 1 and 2.9 log CFU/g range in which some were below the detectable level (Table 4).

### 3.5. Frequency of Isolation of Pathogens

Among 100 vegetable samples analyzed, 11% were contaminated by *S. aureus*. *S. aureus* isolates were highly prevalent in cabbage (20%) followed by lettuce (15%). On the contrary, only 1 (5%) *S. aureus* was isolated from green pepper samples. In carrot samples, *S. aureus* was not detected (Table 5). The present study showed that 15.0% of vegetable samples were positive for *Salmonella* and could be detected in all vegetable types (Table 5). A high prevalence (25%) of *Salmonella* was isolated from lettuce samples followed by tomato and cabbage (20% from each sample). The least (5%) were detected from each green pepper and carrot sample (Table 5). *Shigella* was not detected in any of the vegetable types.

## 4. Discussion

Microorganisms such as bacteria, fungi, and viruses are ubiquitous that may reside on vegetables to survive [24], suggesting the sanitary and hygienic quality of the postharvesting practice, water, transportation, storage, and processing of the production [25–27]. Consumption of raw or partly cooked vegetables is a common practice among the population of the study sites. The present assessment of vegetable samples analyzed for microbiological quality indicated high counts including aerobic mesophilic bacteria, Enterobacteriaceae, coliforms, and Staphylococci. However, the yeast and mold counts showed relatively low counts.

The present study showed that AMB count values were between 3.4 log CFU/g (green pepper) and 7.4 log CFU/g (cabbage). On the contrary, Khiyami et al. [28] reported that AMB counts were between 5 log CFU/g and 5.7 log CFU/g. Other vegetables such as leaf lettuces, spinach, and carrot were also reportedly positive (3.6 log10 CFU/g) for AMB [20]. Mritunjay and Kumar [29] reported that most of these microorganisms managed to grow in the storage temperature. Therefore, high counts of AMB are an indication of exposure to contaminants because of the existence of favorable conditions [30]. Furthermore, domesticated food animals, flies, and rodents might be a source of contamination through direct contact at vegetable farms [31]. Foods containing aerobic mesophilic bacteria are considered as good (<4 log10 CFU/g), average (4.0–6.7log10 CFU/g), poor (6.7–7.7 log10 CFU/g), and spoiled food (>7.7log10 CFU/g) [32]. Based on these criteria, only 10% of green peppers were regarded as good, whereas tomato, carrot, and lettuce were average. But 15% of cabbage samples can be rated as poor of microbial quality regarding aerobic mesophilic bacteria. The samples with the highest Enterobacteriaceae counts were cabbage (6.3 CFU/g) and the lowest from tomato (2.8 log CFU/g). A study conducted by Guchi and Ashenafi [33] at Addis Ababa and a study from Morocco [34] reported a higher Enterobacteriaceae count (>4 log CFU/g) in lettuce and green pepper. Abadias et al. [35] recorded almost 79% of contamination in the samples with Enterobacteriaceae counts (<5 log CFU/g). However, our study showed only 65% of Enterobacteriaceae count with < 5 log CFU/g. Coliforms were also detected from 2.1 log CFU/g (tomato) to 5.9 log CFU/g (cabbage). In Zambia [36], coliforms ranged between 2.2 and 5.9 log CFU/g. A study from Saudi Arabia [28] reported that the coliform counts of salad were from 4.3 to 4.9 log CFU/g. Aycicek et al. [32] also found coliforms from 3.0 to 6.9 log10 CFU/g. Total coliform presence may indicate contamination of vegetables from irrigation water, animal waste, sewage, soil, and feces [37]. Based on the findings of this study, higher counts of Enterobacteriaceae and coliform in cabbage and other vegetable samples are attributed to poor hygiene practices of vegetables during harvesting. Furthermore, the impact of animal manure, unclean storerooms, unsuitable marketplaces, animal transportation, and wastewater irrigation should never be underestimated.

A high count of Staphylococci (4–6.9 log CFU/g) was recorded in cabbage and low (2.0–3.9 log CFU/g) in green pepper and tomato samples. *Staphylococcus* counts between 4.0 and 6.0 log CFU/g [33] were reported in about 80% samples of green pepper and lettuce. Eni et al. [38] showed that *S. aureus* is frequent contamination from vegetables. It has been reported that the production of enterotoxin occurs when the counts of *Staphylococcus aureus* reach 6 log CFU/g [39]. *Staphylococcus aureus* might occur on the vegetables' surface through contact from unwashed hands during choosing of vegetables to buy [40]. The observed mean counts of yeasts and molds were ≤2.9 log CFU/g in the current study of all of the vegetable samples (100%). Moreover, this study indicated that 55% of vegetable samples showed yeast and mold counts below the detectable level. Similar to our results, Dugassa et al. [4] showed that the counts of yeasts and molds in carrot and tomato were undetectable. Mritunjay and Kumar [29] recorded that yeast and mold counts range from 0.3 to 5.5 log cfu/g. Abadias et al. [35] and Tournas [41] obtained similar results with samples of fresh and minimally processed vegetables. In contrast to the present study, Seow et al. [42] isolated more yeast and mold counts from tomatoes as compared to the bacterial count. Some molds can produce mycotoxins and allergens, and large numbers of conidia may induce health instability [41, 43].

The presence of *S. aureus* in vegetables is dangerous to consumers. For instance, *Salmonella* spp. is associated with humans' gastrointestinal disorders, fever, abdominal cramp, vomiting, and diarrhea due to food poisoning [44]. Vegetables may get contaminated through improper handling [45] and from other environmental factors. A study conducted in Arba Minch Town, southern Ethiopia also reported that less hygienically handled vegetables which are usually consumed in raw served to transmit various infectious diseases [27, 44]. Therefore, cleaning using disinfectants should be practiced to make the vegetables fit for consumption in raw form.

In the present study, 25% of lettuce samples were found contaminated with *Salmonella* spp. [57] reported from Jimma city that 20.7% of the samples were positive for *Salmonella*. Another study conducted in the same city by Dugassa et al. (give year of publication) revealed that 16.7% of the lettuce samples have been contaminated with *Salmonella* spp. Rajkowski and Fan [45] reported that *Salmonella* spp. was prevalent particularly from lettuce samples. Folded foliar surfaces of lettuce leaves may have contributed by making a conducive surface for bacterial growth [32, 49, 50]. Kumar [51] and Singh et al. [52] found that vegetables generally harbor *Salmonella* spp. Singh et al. [52] also indicated that stagnant water used for sprinkling and cleaning vegetables might be the most important way of contaminating vegetables with *Salmonella* spp. This report is in confirmation with the finding from the Middle East which detected 6.7% of the raw vegetables from the postharvest, washing areas of Bekaa Valley, Lebanon, and Beirut [53]. In addition, producing vegetables by using manure, using contaminated water for irrigation might have significantly contributed to the contamination [38]. The presence of *Salmonella,* above the recommended limit, could pose a serious threat to the health of consumers and is regarded unacceptable for consumption [54]. To control the contamination of vegetables by microbial pathogens, different techniques such as disinfection [55, 56], modified atmospheric conditions, refrigeration, and innovative technologies need to be exploited before consumption [6] because thorough washing was not reportedly sufficient to reduce pathogen levels to safer limits in leafy vegetable types studied [57].

## 5. Conclusion

In the current study, out of five vegetable types analyzed, cabbage samples were contaminated with high microbial load followed by carrot. Prevalence of *Salmonella* and *Staphylococcus aureus* in cabbage than the other vegetable samples indicate the poor sanitary condition and improper handling at both harvesting and marketing. Detection of pathogens from vegetables necessitates the adoption of best agricultural and handling practices by farmers, and good hygienic practices of food vendors, processors, and consumers are required to minimize the risks of transmission of pathogens. The possible causes of preharvesting and postharvesting contamination of vegetables might be due to their production on contaminated soil and washing of the vegetable with wastewater. The results of the present study, especially the identification of microorganisms, are also useful to the clinicians for treating the patients with the right kind of medicines. Farmers should be informed about the sources of microbial contamination. Furthermore, the government may have a close observation of the pre and postharvesting activities of vegetable producers and sellers to minimize the risk of diseases. Fresh vegetables should be protected from contamination by humans, animals, and other wastes which may constitute a hazard to the health of the consumer through fresh produces. Moreover, it is recommended to avoid consuming raw vegetables without adding food-grade chemicals (antimicrobial agents) which reduce microbial load.

## Figures and Tables

**Table 1:** Sociodemographic characteristics of vegetable cultivars, sellers, retailers, and consumers in Fitche town, Central Ethiopia, 2020.

Characteristics	Frequency	Percent
Address		
Urban	108	90
Rural	12	10
Academic status		
Illiterate	0	0
Elementary education	42	35
Secondary education and above	78	65
Religion		
Muslim	1	0.8
Orthodox	112	93.3
Protestant	7	5.8
Others	0	0
Respondent type		
Cultivar	6	5
Seller	15	12.5
Retailer	27	22.5
Consumer/Buyer	72	60

Number of respondents (*N* = 120).

**Table 2:** General awareness and sanitary conditions of vegetable cultivars, sellers, retailers, and consumers in Fitche town, Central Ethiopia, 2020.

Characteristics	Response	Frequency	Percent
Information about contaminated vegetables can cause diseases to humans	Yes	102	85
	No	18	15
Health problems occurred due to consuming contaminated vegetables	Yes	78	65
	No	42	35
Transportation of fresh vegetables to reach the market	Humans	30	25
	Donkey/Horse	12	10
	Cart	24	20
	Car	54	45
Transporting containers	Sac	64	53
	Plastic bags	18	15
	Boxes	36	30
Organization of vegetables in the market without close contact	Yes	67	56
	No	53	44
Using shade to protect vegetables from physical and solar damage	Yes	69	57.5
	No	51	42.5
Time takes fresh vegetables left with seller/retailer before totally sold to the consumer	<1 day	36	30
	1 day	21	17.5
	2 days	9	7.5
	3 days	48	40
	>3 days	6	5
Addition of food-grade chemicals such as vinegar for raw consumed vegetables	Yes	60	50
	No	60	50
Using a refrigerator to store fresh vegetables	Yes	54	45
	No	66	55

Number of respondents (*N* = 120).

**Table 3:** Microbial counts (log CFU/g) of selected vegetables collected from markets in Fiche town, Central Ethiopia, 2020.

Vegetable types	Microbial groups log *∗*CFU/g (mean ± standard error)					
	AMB^†^	EB^‡^	Coliforms	Staph^§^	Yeast	Mold
Tomato	5.7 ± 0.3	3.7 ± 0.5	2.8 ± 0.5	2.5 ± 0.2	2.3 ± 0.3	2.2 ± 0.4
Cabbage	6.4 ± 0.6	5.7 ± 0.3	5.1 ± 0.5	5.3 ± 0.4	2.4 ± 0.3	2.2 ± 0.3
Green pepper	4.7 ± 0.6	4.7 ± 0.5	3.4 ± 0.5	3.7 ± 0.2	2.1 ± 0.3	1.7 ± 0.3
Carrot	6.2 ± 0.7	4.2 ± 0.5	3.5 ± 0.4	3.3 ± 0.4	2.2 ± 0.2	1.8 ± 0.3
Lettuce	5.3 ± 0.7	5.2 ± 0.8	4.3 ± 0.7	4.0 ± 0.5	2.5 ± 0.3	2.0 ± 0.3
Mean (*N* = 100)	5.7 ± 0.6	4.7 ± 0.5	3.8 ± 0.5	3.8 ± 0.3	2.3 ± 0.3	2.0 ± 0.3

^†^Aerobic mesophilic bacteria, ^‡^Enterobacteriaceae, ^§^Staphylococci, and *∗*a colony forming unit.

**Table 4:** Frequency distribution of tested microbes of selected vegetables sold in Fitche town, Central Ethiopia, 2020.

Microbial groups	Sample type	*∗*Log CFU/g (%)						
		<2	2–2.9	3–3.9	4–4.9	5–5.9	6–6.9	7–7.9
AMB^†^	Tomato	—	—	—		17 (85)	3 (15)	
	Cabbage				2 (10)	4 (20)	11 (55)	3 (15)
	Green pepper	—	—	2 (10)	13 (65)	5 (25)	—	—
	Carrot	—	—	—	2 (10)	4 (20)	14 (70)	—
	Lettuce	—	—	—	4 (20)	13 (65)	3 (15)	—

EB^‡^	Tomato	—	1 (5)	15 (75)	4 (20)	—	—	—
	Cabbage	—	—	—	—	17 (85)	3 (15)	—
	Green pepper	—	—	3 (15)	11 (55)	6 (30)	—	—
	Carrot	—	—	9 (45)	10 (50)	1 (5)	—	—
	Lettuce	—	—	—	8 (40)	7 (35)	5 (25)	—

Coliforms	Tomato	—	12 (60)	8 (40)	—	—	—	—
	Cabbage	—	—	—	6 (30)	14 (70)	—	—
	Green pepper	—	4 (20)	15 (75)	1 (5)	—	—	—
	Carrot	—	2 (10)	16 (80)	2 (10)	—	—	—
	Lettuce	—	—	3 (30)	13 (65)	4 (20)	—	—

Staph^§^	Tomato	—	20 (100)	—	—	—	—	—
	Cabbage	—	—	—	3 (15)	16 (80)	1 (5)	—
	Green pepper	—	—	20 (100)	—	—	—	—
	Carrot	—	2 (10)	16 (80)	2 (10)	—	—	—
	Lettuce	—	—	8 (40)	12 (60)	—	—	—

Yeasts	Tomato	4 (20)	16 (80)	—	—	—	—	—
	Cabbage	1 (5)	18 (90)	1 (5)	—	—	—	—
	Green pepper	5 (25)	15 (75)	—	—	—	—	—
	Carrot	4 (20)	16 (80)	—	—	—	—	—
	Lettuce	—	20 (100)	—	—	—	—	—

Mold	Tomato	4 (20)	16 (80)	—	—	—	—	—
	Cabbage	4 (20)	16 (80)	—	—	—	—	—

^†^Aerobic mesophilic bacteria, ^‡^Enterobacteriaceae, ^§^Staphylococci, and *∗*a colony forming unit.

**Table 5:** Frequency of isolation of *S. aureus, Salmonella* spp, and *Shigella* spp of some vegetables sold in Fitche town, Central Ethiopia, 2020.

Vegetable types	Sample size (100)	No. of *S. Aureus* (%)	No. of *Salmonella* spp. (%)
Tomato	20	10.0	20.0
Cabbage	20	20.0	20.0
Green pepper	20	5.0	5.0
Carrot	20	0.0	5.0
Lettuce	20	15.0	25.0
Total	100	11.0	15.0

## Data Availability

The data used to support the findings of this study are included within the article.
